# PDCL3 is a prognostic biomarker associated with immune infiltration in hepatocellular carcinoma

**DOI:** 10.1186/s40001-024-01787-7

**Published:** 2024-03-17

**Authors:** Wenzhi Jin, Ganggang Wang, Meiyuan Dong, Xiaoliang Wang

**Affiliations:** 1 Department of Hepatobiliary Surgery, Pudong Hospital Affiliated to Fudan University, 2800 Gongwei Road Pudong, Shanghai, 201399 People’s Republic of China; 2 Department of Endocrinology, Pudong Hospital Affiliated to Fudan University, 2800 Gongwei Road Pudong, Shanghai, 201399 People’s Republic of China

**Keywords:** PDCL3, Hepatocellular carcinoma, Prognosis, Biomarker, Immune infiltration

## Abstract

**Background:**

Phosducin-like 3 (PDCL3) is a member of the photoreceptor family, characterized by a thioredoxin-like structural domain and evolutionary conservation. It plays roles in angiogenesis and apoptosis. Despite its significance, research on the biological role of PDCL3 in liver hepatocellular carcinoma (LIHC) remains limited. This study aims to explore the prognostic value and potential mechanisms of PDCL3 in cancer, particularly in LIHC, through bioinformatics analysis.

**Methods:**

RNA-seq data and corresponding clinical information for pan-cancer and LIHC were extracted from the TCGA database to analyze PDCL3 expression and survival prognosis. Differential expression of PDCL3 was analyzed using the HPA database. GO and KEGG enrichment analysis were performed for PDCL3-associated genes. The relationship between PDCL3 expression and various immune cell types was examined using the TIMER website. Clinical samples were collected, and immunohistochemistry and immunofluorescence experiments were conducted to validate the differential expression of PDCL3 in LIHC and normal tissues. In vitro assays, including CCK-8, wound healing, Transwell, and colony formation experiments, were employed to determine the biological functions of PDCL3 in LIHC cells.

**Results:**

Analysis from TIMER, GEPIA, UALCAN, and HPA databases revealed differential expression of PDCL3 in various tumors. Prognostic analysis from GEPIA and TCGA databases indicated that high PDCL3 expression was associated with poorer clinical staging and prognosis in LIHC. Enrichment analysis of PDCL3-associated genes revealed its involvement in various immune responses. TCGA and TIMER databases showed that high PDCL3 expression in LIHC decreased tumor immune activity by reducing macrophage infiltration. PDCL3 exhibited positive correlations with multiple immune checkpoint genes. Immunohistochemistry (IHC) and immunofluorescence (IF) experiments confirmed elevated PDCL3 expression in LIHC tissues compared to adjacent normal tissues. In vitro experiments demonstrated that PDCL3 promoted LIHC cell proliferation, migration, invasion, and colony-forming ability.

**Conclusion:**

PDCL3 is highly expressed in various cancer types. Our study suggests that elevated PDCL3 expression in hepatocellular carcinoma is associated with poorer prognosis and may serve as a potential diagnostic biomarker for LIHC. PDCL3 may regulate the biological functions of LIHC by modulating immune infiltration. However, the precise regulatory mechanisms of PDCL3 in cancer warrant further investigation.

## Introduction

Cancer continues to pose a significant global public health challenge [1–3], inflicting immense suffering on patients due to its high recurrence, metastasis, and other formidable factors [4]. Various cancer treatment modalities, including surgery, radiation therapy, targeted therapy, and immunotherapy, have advanced our understanding of the complex mechanisms underlying tumorigenesis, thereby improving therapeutic outcomes [5]. In this context, bioinformatics methods have gained widespread application in cancer research, offering advantages such as large sample sizes, cost-effectiveness, and efficiency. Pan-cancer analyses, which involve the examination of genes across various cancer types and the identification of differentially expressed genes, have proven valuable in cancer diagnosis, prognosis, and immunotherapy, leveraging publicly available databases.

Phosducin-like 3 (PDCL3) belongs to the Phosducin-like protein (PhLP) family, characterized by an N-terminal helical domain, a central thioredoxin-like domain, and a negatively charged carboxyl-terminal region [6]. PhLP3 plays a pivotal role in maintaining β-tubulin levels in mammalian cells and participates in cell cytoskeletal remodeling through mechanisms associated with the MAPK pathway and RhoA-dependent processes [7]. Additionally, PDCL3 enhances pathological angiogenesis by increasing tyrosine phosphorylation induced by vascular endothelial growth factor (VEGF), playing a central role in tumor growth. Thus, PDCL3 is considered an essential gene in promoting tumor growth and proliferation.

However, the precise role of PDCL3 in cancer remains unclear. In this study, we employed the Cancer Genome Atlas (TCGA), UALCAN, and other databases to investigate PDCL3 expression in pan-cancer and conducted an in-depth exploration of its expression, clinical relevance, prognostic impact, and functional implications in LIHC. Subsequently, we validated the differential expression of PDCL3 in normal and LIHC tissues and explored its influence on the biological behaviors of LIHC cells.

## Materials and methods

### Data collection and processing

Level 3 HTSeq-FPKM format RNA-seq data and corresponding clinical information were retrieved from the ALL (pan-cancer) project within The Cancer Genome Atlas (TCGA) (https://portal.gdc.cancer.gov/). The RNA-seq data in FPKM format were log2-transformed for analysis. Specifically, RNA-seq data for Liver Hepatocellular Carcinoma (LIHC) and its clinical information were extracted for subsequent R package analysis.

### PDCL3 expression analysis

PDCL3 expression in multiple tumor tissues and adjacent normal tissues was analyzed using various databases, including TIMER 2.0 (http://www.cistrome.shinyapps.io), Gene Expression Profiling Interactive Analysis (GEPIA) (http://gepia.cancer-pku.cn/detail.php), and UALCAN (http://ualcan.path.uab.edu). RNA-seq data (Level 3) and corresponding pan-cancer clinical information were obtained from TCGA (https://portal.gdc.com). Statistical analysis was conducted using R software version 4.0.3 and ggplot2 (version 3.3.2). A *p *value less than 0.05 was considered statistically significant. To validate the differential expression of PDCL3 in normal and liver cancer tissues, Immunohistochemistry (IHC) images downloaded from the Human Protein Atlas (HPA) (http://www.proteinatlas.org) for normal and tumor tissues of liver cancer samples were analyzed.

### Prognostic analysis

We utilized the GEPIA database to analyze the survival prognosis of relevant tumors. Additionally, clinical information extracted from TCGA databases for the respective tumors was analyzed for survival prognosis. Kaplan–Meier survival curves, including overall survival (OS), progression-free survival (PFS), recurrence-free survival (RFS), and disease-specific survival (DSS), were plotted using the Kaplan–Meier Plotter (http://kmplot.com). Time-dependent receiver operating characteristic (ROC) analysis was performed to compare the predictive accuracy and risk score of the PDCL3 gene. Bar charts were created using the “rms” package to predict the 1-year and 3-year overall recurrence rates. Forest plots provided graphical results of these factors for calculating individual prognosis risk based on the score associated with each risk factor.

### Immune infiltration analysis

The relationship between PDCL3 expression and immune infiltration in hepatocellular carcinoma was analyzed using the TIMER2 tool. We evaluated the gene expression's association with immune infiltration using various algorithms, including TIMER, TIDE, CIBERSORT, CIBERSORT-ABS, QUANTISEQ, XCell, MCP Counter and EPIC algorithms. Reliable immune score evaluations were conducted on data extracted from TCGA databases using the R package Immunedeconv, which integrates six advanced algorithms: TIMER, xCell, MCP-counter, CIBERSORT, EPIC, and quanTIseq. Systematic benchmarking of these algorithms showed their unique performance advantages. Genes such as SIGLEC15, TIGIT, CD274, HAVCR2, PDCD1, CTLA4, LAG3, and PDCD1LG2 were related to immune checkpoint genes. The expression of these eight genes was assessed to evaluate the expression of immune checkpoint-related genes. All these analyses were implemented using R (v4.0.3) software packages ggplot2 and pheatmap.

### PDCL3-related enrichment analysis

We employed Gene Ontology (GO), Kyoto Encyclopedia of Genes and Genomes (KEGG), and Gene Set Enrichment Analysis (GSEA) to predict the biological pathways and molecular functions associated with PDCL3 in liver cancer, elucidating the oncogenic roles of target genes. LIHC data extracted from TCGA based on PDCL3 expression were processed and divided into high expression and low expression groups. Differential expression analysis was conducted, and volcano plots were generated for both groups. Enrichment analysis was performed on the differentially expressed genes (DEGs). All these analyses were conducted using R (v4.0.3) software packages ggplot2 and pheatmap.

### Reagents and instruments

Microplate Reader (Thermo Fisher, USA); ChemiDoc Imaging System (BIORAD, USA); Reverse Transcriptase (Wuhan Miaoling Biotechnology Co., Ltd.); RIPA Lysis Buffer, BCA Protein Assay Kit (Shanghai Epizyme Biotechnology Co., Ltd.); ECL Chemiluminescent Substrate (Milipore, USA); Cell Counting Kit8 Cell Proliferation Assay Kit (Suzhou UElandy Biotechnology Co., Ltd.); Crystal Violet Staining Solution (Shanghai Beyotime Biotechnology Co., Ltd.); Transwell Chambers (Corning, USA); Rabbit Anti-Human PDCL3 Monoclonal Antibody (Proteintech, USA); Mouse Anti-Human GAPDH Polyclonal Antibody (Proteintech, USA); HRP-conjugated Goat Anti-Rabbit IgG Secondary Antibody (Shanghai Beyotime Biotechnology Co., Ltd.); HRP-conjugated Goat Anti-Mouse IgG Secondary Antibody (Shanghai Beyotime Biotechnology Co., Ltd.)

### Clinical sample collection

Hematoxylin–eosin (HE) stained slides and paraffin-embedded histological samples were retrospectively collected from 46 liver cancer patients who underwent liver resection at Fudan University Affiliated Pudong Hospital between January 1, 2016, and December 31, 2022. None of the patients had received radiotherapy or chemotherapy prior to the surgery. Tissue microarrays (TMA) were constructed from these samples, including 46 primary liver hepatocellular carcinoma tissues and matched adjacent normal liver tissues. The study was approved by the ethics committee of Fudan University Affiliated Pudong Hospital, and written informed consent was obtained from all patients.

### Cell culture and transfection

Human liver cancer cell lines HepG2, Hep3B, 97-H, Huh-7, and the human liver immortalized cell line THLE-2 originated from the Chinese Academy of Medical Sciences Cancer Cell Repository. These cells were cultured in DMEM containing 10% fetal bovine serum (FBS) and maintained in a cell culture incubator at 37 °C with a CO_2_ concentration of 5%. Cell growth and confluence were observed under a microscope, and when confluence reached 80–90%, cells were passaged at a ratio of 1:4. Stable cell lines were constructed using a retrovirus as a vector.

### Immunohistochemistry and immunofluorescence

For immunohistochemistry, tissue cores underwent sequential steps of deparaffinization, rehydration, blocking, antigen retrieval, and cooling to room temperature. Primary antibodies were applied, followed by overnight incubation at 4 °C. Subsequently, secondary antibodies were added, followed by three washes with PBS. DAB staining, counterstaining, and fixation were performed. Five random fields were selected under a microscope, and the presence of brown particles in the cell nucleus and cytoplasm was defined as positive. The percentage of positive cells was scored as follows: 0 (no positive cells), 1 (< 25% positive), 2 (25–50% positive), 3 (50–75% positive), and 4 (> 75% positive). The Immunoreactive Score (IRS score) was calculated as = staining intensity multiplied by the percentage of positive cells [8]. The H-score was calculated as the sum of (percentage of weak staining × 1) + (percentage of moderate staining × 2) + (percentage of strong staining × 3) [9, 10].

For immunofluorescence, tissue cores were immersed in deacetylation solution for 30 min, followed by ethanol dehydration in a graded series. The slides were then immersed in antigen retrieval solution and microwaved for 25 min, followed by cooling to room temperature. Tissue sections were blocked with a solution containing 5% goat serum at room temperature for 1 h. Primary antibodies were added and incubated overnight at 4 °C. Secondary antibodies were added and incubated for 1 h at room temperature. DAPI solution was used for staining for 20 min before mounting the slides.

### Western blot

Following transfection, cells from the knockdown group, overexpression group, and control group were cultured in a six-well plate until they reached 100% confluence. Protein extraction was performed using high-intensity RIPA lysis buffer, followed by centrifugation at 12,000 rpm for 20 min at 4 °C to collect the supernatant. The protein concentration was quantified using the BCA method. Proteins were separated by SDS–PAGE electrophoresis at a constant voltage of 120V for 90 min. Subsequently, proteins were transferred onto a PVDF membrane with a pore size of 0.45 μm using a wet transfer system at a constant current of 400 mA for 60 min. The PVDF membrane was then blocked with 5% skimmed milk (diluted in TBST, Tris-buffered saline with Tween 20) for 1 h at room temperature. Primary antibodies against rabbit anti-human PDCL3 (1:1000) and β-tubulin (1:1000) were added and incubated. After incubation, the PVDF membrane was washed with TBST for 5 min, repeated three times. HRP-conjugated secondary antibodies (1:3000) were then added and incubated for 1 h at room temperature. The membrane was washed with TBST for 5 min, repeated three times. ECL luminescent reagent was applied, and the membrane was exposed using a gel imaging system in a light-protected environment. The protein bands of interest were analyzed using Image J software.

### Cell viability assay

Transfected knockdown, overexpression, and control cells were seeded in a 96-well plate at a density of 3000 cells per well. Each well was supplemented with 100 µl of complete culture medium. After 24, 48, and 72 h of incubation, the original culture medium was removed, and 100 µl of DMEM medium containing 10% CCK-8 reagent was added to each well. The cells were then incubated in a light-protected environment at 37 °C with 5% CO_2_ for 1 h. Absorbance at 450 nm was measured using a microplate reader. The cell proliferation rate was calculated using the formula:

$${\text{Cell}}\;{\text{proliferation}}\;{\text{rate}} =\, \left( {{\text{OD}}\;{\text{experimental}}\;{\text{group}} - {\text{OD}}\;{\text{blank}}\;{\text{well}}} \right)/\left( {{\text{OD}}\;{\text{control}}\;{\text{group}} - {\text{OD}}\;{\text{blank}}\;{\text{well}}} \right) \times {1}00\% .$$

### Transwell invasion analysis

Transwell chambers were placed in a 24-well plate, and 100 µl of Matrigel was evenly coated on the upper chamber. The chambers were allowed to stand for 2 h in a cell culture incubator. Transfected cells were harvested, digested, and centrifuged. The cells were resuspended in DMEM medium (density of 2.5 × 10^5^ cells/ml). Subsequently, 200 µl of cell suspension was added to the upper chamber, and 500 µl of DMEM medium containing 10% fetal bovine serum was added to the lower chamber. The cells were incubated in a cell culture incubator at 37 °C with 5% CO_2_ for 48 h. After removing the culture medium, cells were fixed with 4% paraformaldehyde solution at room temperature for 20 min. They were then stained with 1% crystal violet dye for 20 min. After washing with PBS, pictures were taken under a microscope, and the invaded cells were counted.

### Cell migration assay

Transfected cells were seeded in a six-well plate at a density of 5 × 10^5^ cells per well. When the cells reached 100% confluence, the culture medium was removed. Using a 200 µl pipette tip, a vertical scratch was made at the bottom of the six-well plate. The plate was washed with PBS three times, and then DMEM medium containing 2% fetal bovine serum was added. Images of the cell scratch were captured at 0, 24, and 48 h under a microscope. Cell migration rate was calculated using the following formula:

$${\text{Cell}}\;{\text{migration}}\;{\text{rate}} =\, \left( {{\text{Width}}\;{\text{of}}\;{\text{scratch}}\;{\text{at}}\;0\,{\text{h}} - {\text{Width}}\;{\text{of}}\;{\text{scratch}}\;{\text{at}}\;{24}\;{\text{or}}\;{48}\,{\text{h}}} \right)/{\text{Width}}\;{\text{of}}\;{\text{scratch}}\;{\text{at}}\;0\,{\text{h}} \times {1}00\% .$$

### Cell colony formation assay

Transfected cells (500 cells/well) were seeded in a six-well plate and incubated at 37 °C with 5% CO_2_ in a cell culture incubator for 10 days. The culture medium was removed, and cells were fixed with 4% paraformaldehyde solution at room temperature for 20 min. Then, cells were stained with 1% crystal violet dye for 20 min. After washing with PBS and drying, digital camera images were taken, and the number of cell colonies formed was counted.

### Statistical analysis

Million transcripts per million (TPM) values were standardized by log2 transformation (1 + TPM). The *t* test was used for comparing two groups. ROC curves were plotted, and the area under the curve (AUC) was calculated using the R package “ROCR.” Kaplan–Meier (KM) estimates and the log-rank test were used for survival analysis. All statistical analyses were performed using R software (v4.0.3). Spearman correlation analysis was used to assess the statistical correlation between two variables. Normally distributed continuous data were presented as mean ± standard deviation ($\overline{x} \pm s$). Chi-squared tests were used for analyzing categorical data. A *p* value of < 0.05 was considered statistically significant.

## Results

### PDCL3 expression and prognostic analysis in pan-cancer

**Fig. 1:**
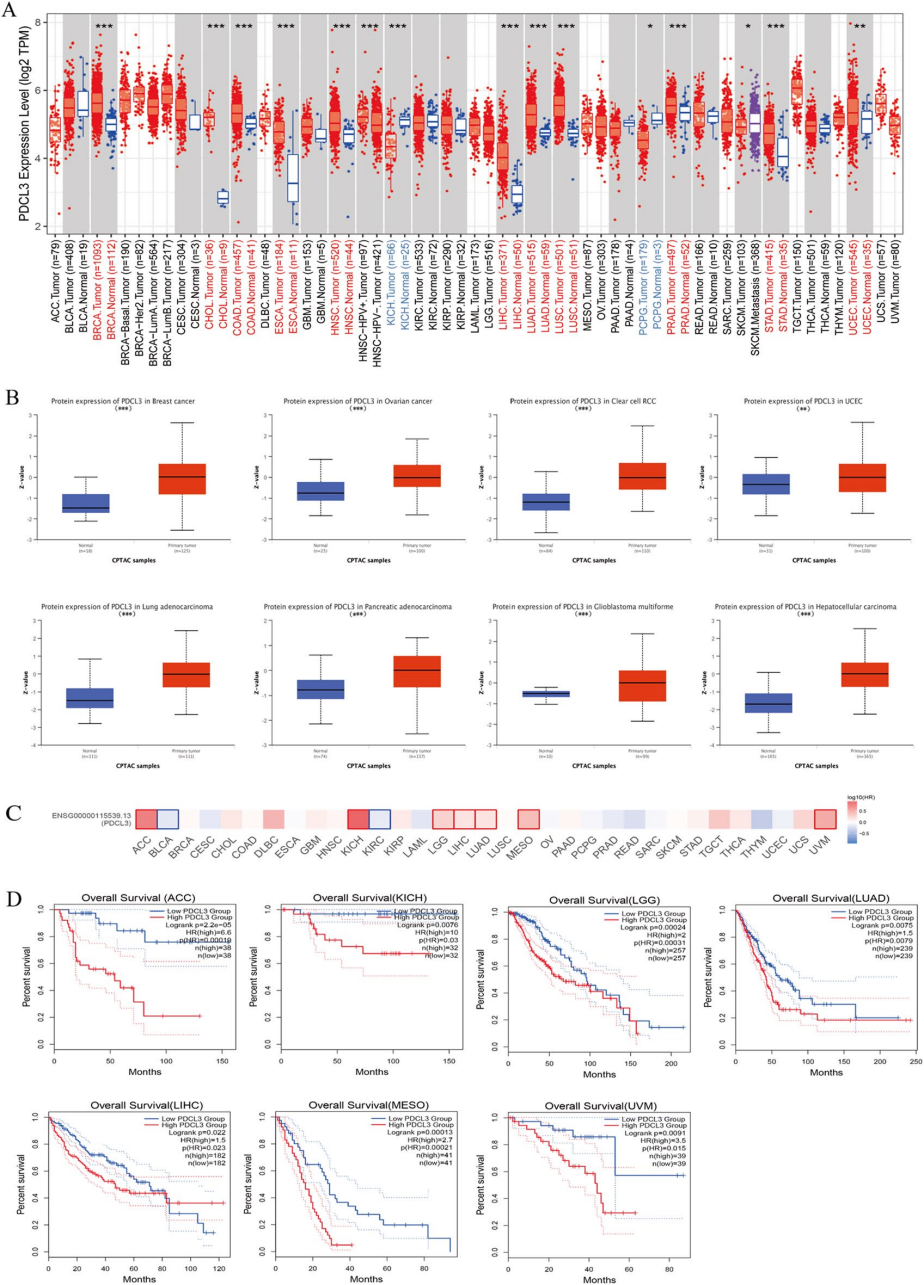
Expression of PDCL3 in pan-cancer tissues. **A** Analysis of PDCL3 expression in various cancers using the TIMER database. **B** Analysis of the differential protein expression of PDCL3 in tumors and normal tissues using the CPTAC database. **C** Expression of PDCL3 in different cancers and its association with prognosis. **D** Kaplan–Meier survival curves for patients with different types of cancer. *p value < 0.05; **p value < 0.01; ***p value < 0.001 (Student’s *t* test)

Pan-cancer gene expression analysis of PDCL3 was conducted using various databases. PDCL3 expression was found to be significant across multiple cancer types in the TIMER database, including BRCA, CHOL, COAD, ESCA, HNSC, LIHC, LUAD, PRAD, STAD, and UCEC (Fig. 1A). Additionally, analysis using the CPTAC dataset in UALCAN revealed elevated PDCL3 protein expression in breast cancer, ccRCC, UCEC, LUAD, PAAD, and GBM (Fig. 1B). Prognostic analysis using GEPIA indicated that high PDCL3 expression correlated with worse prognosis in various cancer types, including ACC, KICH, LGG, LUAD, LIHC, MESO, and UVM (Fig. 1C). Kaplan–Meier curves further supported these findings (Fig. 1D).

### Expression of PDCL3 in LIHC

**Fig. 2:**
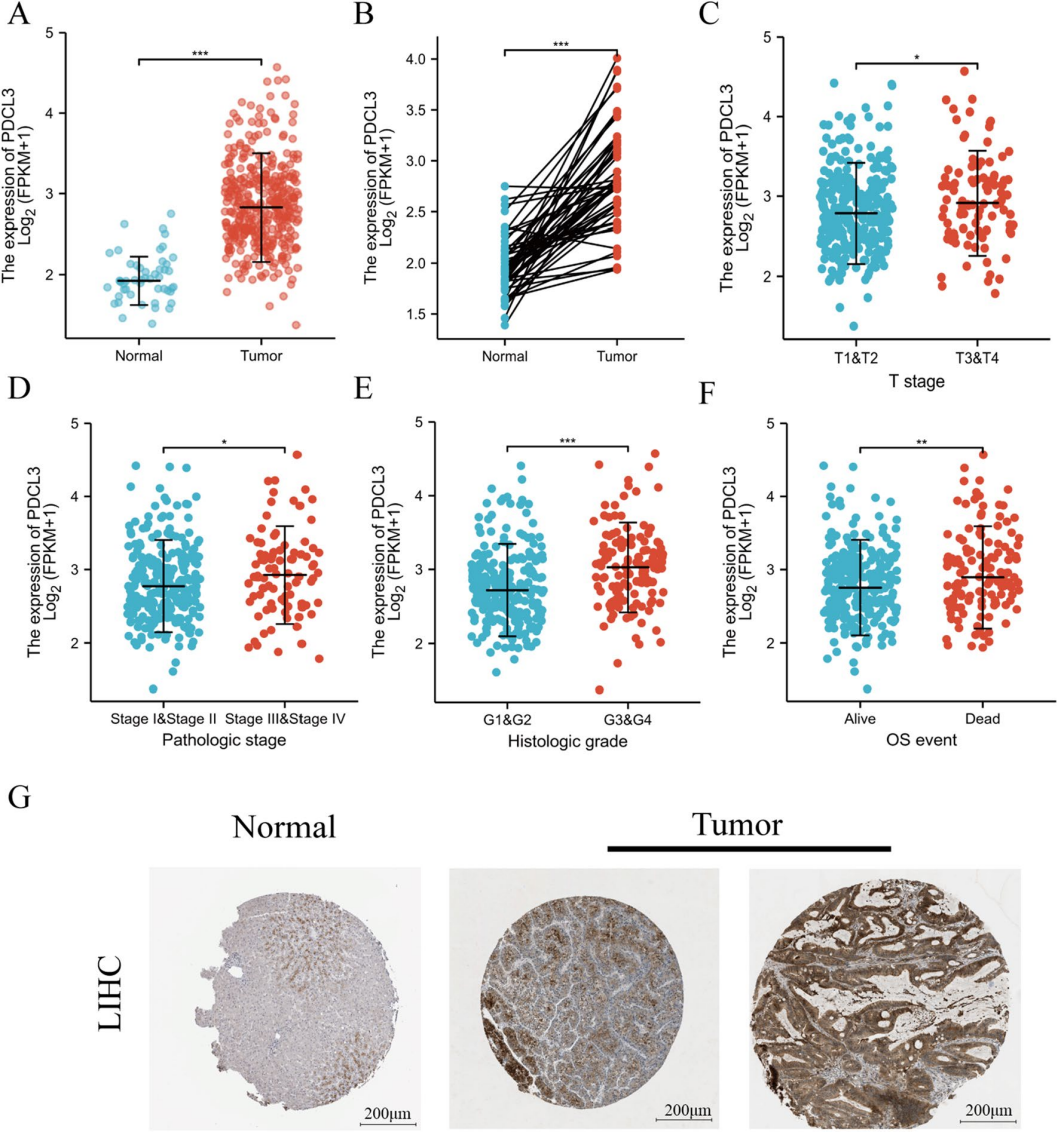
Overexpression of PDCL3 in LIHC. **A** Expression levels of PDCL3 in LIHC tumor tissues and normal tissues. **B** Expression levels of PDCL3 in LIHC (hepatocellular carcinoma) and matched adjacent normal tissues. **C**–**E** Relationship between PDCL3 overexpression and clinical, pathological, and histological staging. **F** Correlation of PDCL3 with overall survival rate. **G** PDCL3 staining from IHC results provided by the HPA database. *p value < 0.05; **p value < 0.01; ***p value < 0.001 (Student’s *t* test)

In the above studies, we found that PDCL3 was highly expressed in LIHC, as confirmed by data from various databases such as TIMER, GEPIA, and UALCAN, and that PDCL3 was associated with the prognosis of LIHC. Subsequently, we analyzed RNA-seq data (Level 3) and corresponding clinical information for 374 cases of LIHC and 50 cases of adjacent normal tissues from the TCGA database (Table 1). The results showed that PDCL3 expression in tumor tissues was significantly higher than in normal tissues (Fig. 2A). This result was validated in LIHC and matched adjacent normal tissues (Fig. 2B). The study also analyzed the expression of PDCL3 in different subgroups and indicated that high PDCL3 expression was associated with worse clinical, pathological, and stage conditions (Fig. 2C–E). PDCL3 expression also varied with different survival statuses, with high PDCL3 expression associated with a worse prognosis (Fig. 2F). We also analyzed the IHC results provided by the HPA database. PDCL3 staining in normal liver tissues was negative or moderate, while it was moderate or intense in tumor tissues (Fig. 2G).

**Table 1:** Baseline characteristics of liver cancer patients (analysis of variance)

Characteristics	Low expression of PDCL3	High expression of PDCL3	*p*
T stage, *n* (%)			**0.009**
T1	107 (28.8%)	76 (20.5%)	
T2	38 (10.2%)	57 (15.4%)	
T3	34 (9.2%)	46 (12.4%)	
T4	5 (1.3%)	8 (2.2%)	
N stage, *n* (%)			0.622
N0	125 (48.4%)	129 (50%)	
N1	1 (0.4%)	3 (1.2%)	
M stage, *n* (%)			0.624
M0	129 (47.4%)	139 (51.1%)	
M1	1 (0.4%)	3 (1.1%)	
Pathologic stage, *n* (%)			**0.017**
Stage I	101 (28.9%)	72 (20.6%)	
Stage II	37 (10.6%)	50 (14.3%)	
Stage III	35 (10%)	50 (14.3%)	
Stage IV	2 (0.6%)	3 (0.9%)	
Histologic grade, *n* (%)			**< 0.001**
G1	33 (8.9%)	22 (6%)	
G2	105 (28.5%)	73 (19.8%)	
G3	43 (11.7%)	81 (22%)	
G4	3 (0.8%)	9 (2.4%)	
Age, median (IQR)	64 (53.5, 70)	59 (50.25, 67.75)	**0.011**

Numbers with *p*-values less than 0.05 are in bold

### Prognostic value of PDCL3 in liver cancer

**Fig. 3:**
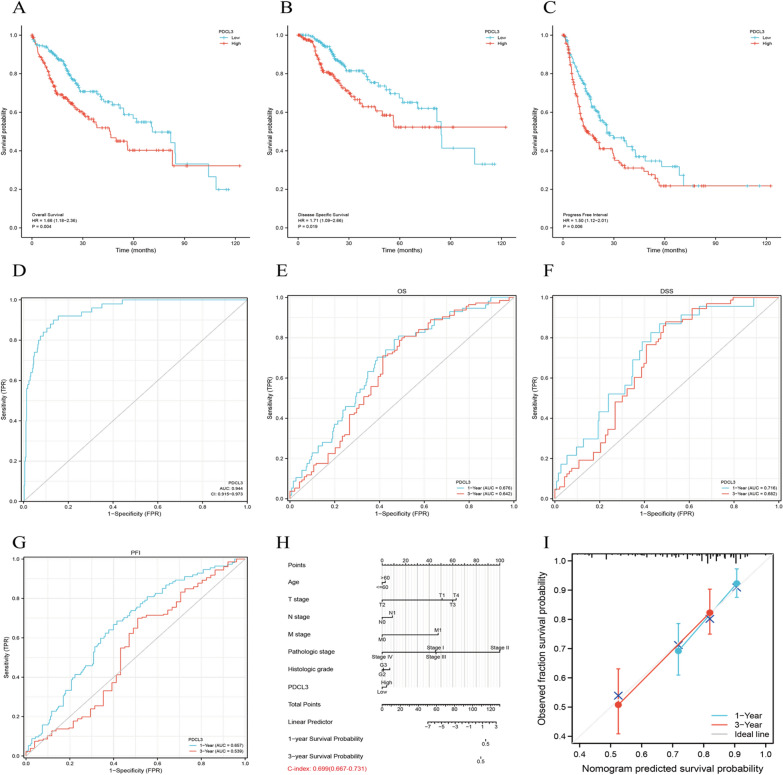
Prognostic relevance of PDCL3 in LIHC. **A**–**C** Relationship between PDCL3 expression and patient prognosis in terms of OS (overall survival), DSS (disease-specific survival), and PFS (progression-free survival). **D** ROC curves for PDCL3 and LIHC prognosis. **E**–**G** Time-dependent ROC curves for PDCL3 and LIHC patient prognosis. **H** Clinical prognostic risk score for PDCL3. **I** Calibration plot for model accuracy assessment

The correlation between PDCL3 expression and the prognosis outcomes of patients with OS, DSS, and PFS is shown in Fig. 3A–C. High PDCL3 expression was associated with poor OS, DSS, and PFS (*p* < 0.05). Furthermore, we used ROC curve analysis to assess the diagnostic value of PDCL3. The results showed an area under the curve (AUC) of 0.944, indicating that PDCL3 might be a potential biomarker for diagnosing LIHC (Fig. 3D). We then plotted time-related ROC curves to evaluate the predictive effect of PDCL3 on different prognosis outcomes of LIHC patients. PDCL3 was found to be effective in predicting 1-year and 3-year OS, DSS, and PFS rates (Fig. 3E–G). We constructed a clinical prognostic risk score for PDCL3 (Fig. 3H). At the same time, the accuracy of the model was assessed using calibration plots (Fig. 3I). The results showed that PDCL3 expression levels could effectively predict 1-year and 3-year OS (*C*-index = 0.699).

### Functional enrichment analysis of PDCL3 in liver cancer

**Fig. 4:**
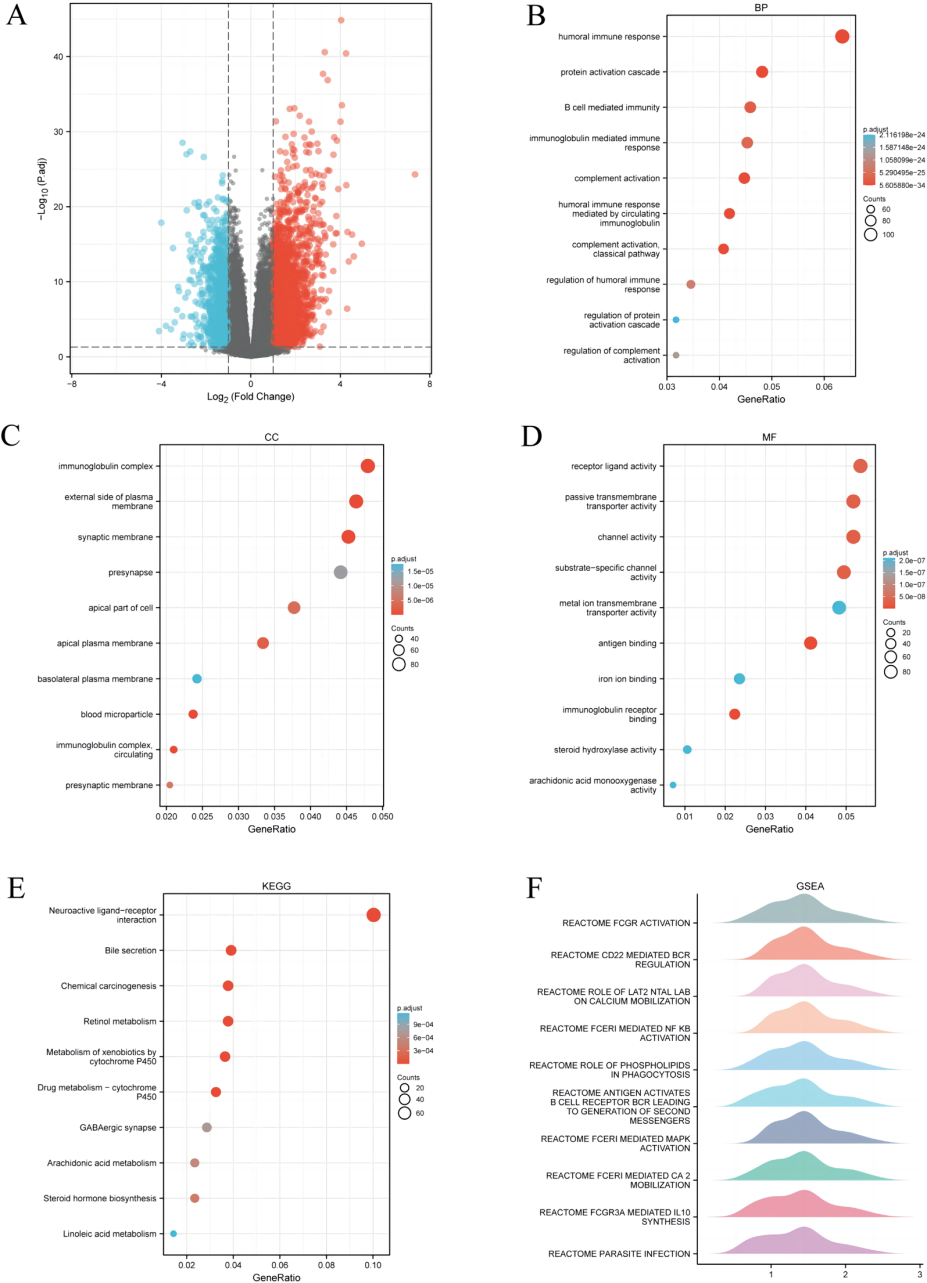
Functional enrichment analysis of PDCL3 in LIHC. **A** Volcano plot of molecules related to PDCL3 expression in LIHC. **B**–**D** Analysis results of BP (biological process), CC (cellular component), and MF (molecular function) related to molecules associated with PDCL3 in LIHC. **E** Pathway analysis related to PDCL3 in LIHC. **F** Hill plot of pathway enrichment analysis related to PDCL3

Subsequently, we conducted PDCL3-related gene expression profiling to further explore the biological functions of PDCL3 in LIHC. After removing null IDs, a total of 34,800 IDs remained. Among them, 3405 IDs met the threshold of |log2(FC)|> 1 and *p*.adj < 0.05, with 2485 being upregulated (positive logFC) and 920 downregulated (negative logFC). The volcano plot was then generated based on these 3405 molecules (Fig. 4A). Furthermore, we performed GO and KEGG enrichment analysis based on the expression of PDCL3. GO analysis showed that the biological functions of PDCL3 genes were mainly related to humoral immune response, protein activation cascade, B-cell-mediated immunity, immunoglobulin complexes, plasma membrane, receptor ligand activity, and passive transmembrane transporter activity (Fig. 4B–D). KEGG enrichment analysis indicated that the neuroactive ligand-receptor interaction and bile secretion pathways were significantly enriched (Fig. 4E). Then, we further conducted GSEA analysis based on PDCL3 expression and generated the corresponding mountain plots (Fig. 4F). GSEA analysis revealed that the high expression phenotype of PDCL3 was mainly involved in FCGR activation and CD22-mediated BCR regulation. In summary, we found a significant enhancement in immune response activity, suggesting that PDCL3 functions are associated with the immune system.

### PDCL3 expression and its relationship with immune infiltration in LIHC

**Fig. 5:**
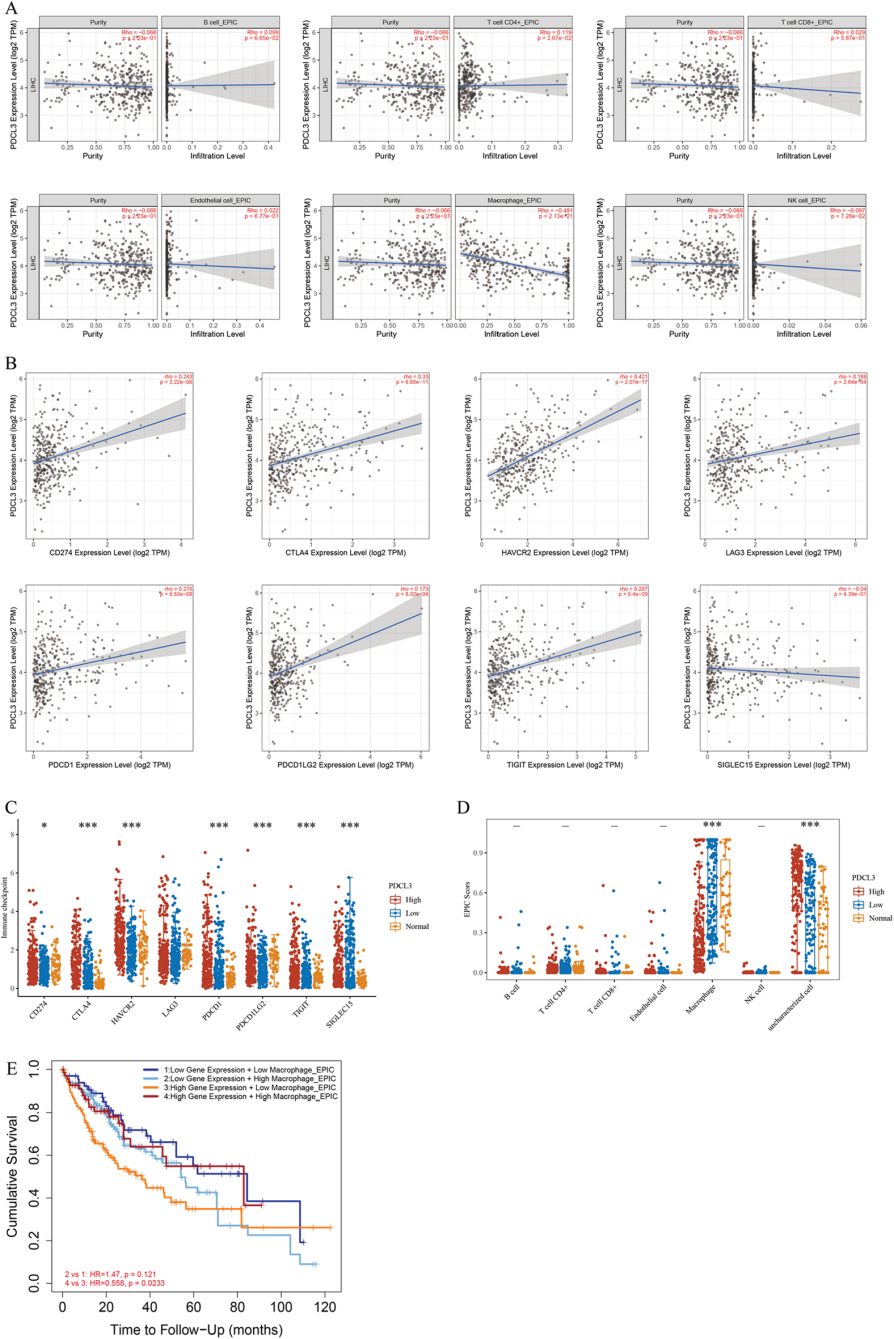
Relationship between PDCL3 expression and immune infiltration in LIHC. **A** Relationship between PDCL3 expression and macrophage infiltration. **B** Correlation of PDCL3 expression with the expression of various immune checkpoint genes in LIHC. **C** Expression levels of individual immune checkpoint genes in different PDCL3 expression groups. **D** Infiltration of immune cells in different PDCL3 expression groups. **E** Analysis of the correlation between high PDCL3 expression, immune infiltration, and adverse prognosis in LIHC patients. *p value < 0.05; **p value < 0.01; ***p value < 0.001 (analysis of variance)

Exploring the gene expression profile of PDCL3 in LIHC, it was found that PDCL3 has biological functions related to the immune system. Subsequently, the relationship between PDCL3 and immune infiltration was further investigated. The TIMER 2.0 database, based on the EPIC algorithm and purity-adjusted data sets, was used to determine the correlation between PDCL3 expression and different immune cell types. The results showed a negative correlation between PDCL3 expression and macrophages (Rho = − 0.481, *p* = 2.13e − 21), while it was unrelated to other immune cells (Fig. 5A). Furthermore, differences in tumor-infiltrating immune cell abundance were determined between high and low PDCL3 expression groups. Macrophage infiltration was significantly lower in the high PDCL3 expression group compared to the low PDCL3 and normal expression groups, but the high PDCL3 group showed significantly higher infiltration of uncharacterized cells. Infiltration of other immune cells did not show significant differences (Fig. 5D).

Further prognostic analysis was performed through Kaplan–Meier (KM) survival curves on immune cell subgroups, aiming to investigate the link between PDCL3 expression and adverse prognosis in LIHC patients, and to determine if high PDCL3 expression is associated with immune infiltration. The results unveiled that in cases with high PDCL3 expression, a decrease in macrophages was indicative of an adverse prognosis, while no significant difference was observed in cases of low PDCL3 expression (Fig. 5E). These findings suggest that high PDCL3 expression contributes to tumor progression and adverse prognosis in LIHC, partly attributed to the decrease in macrophages.

Subsequently, we investigated the correlation between PDCL3 and various immune checkpoint markers in LIHC. Notably, PDCL3 exhibited a positive correlation with CD274 (cor = 0.243, *p* = 2.22e − 06), CTLA4 (cor = 0.33, *p* = 6.85e − 11), HAVCR2 (cor = 0.421, *p* = 0.00e + 00), PDCD1 (cor = 0.276, *p* = 6.53e − 08), and TIGIT (cor = 0.297, *p* = 5.40e − 09) (Fig. 5B). Likewise, we assessed the expression of immune checkpoint genes in high PDCL3, low PDCL3, and normal expression groups. The results revealed differential expression among the three groups for CD274, CTLA4, HAVCR2, PDCD1, PDCD1LG2, TIGIT, and SIGLEC15; however, no significant difference was observed for LAG3 (Fig. 5C).

### Differential expression of PDCL3 in cancer and adjacent tissues

**Fig. 6:**
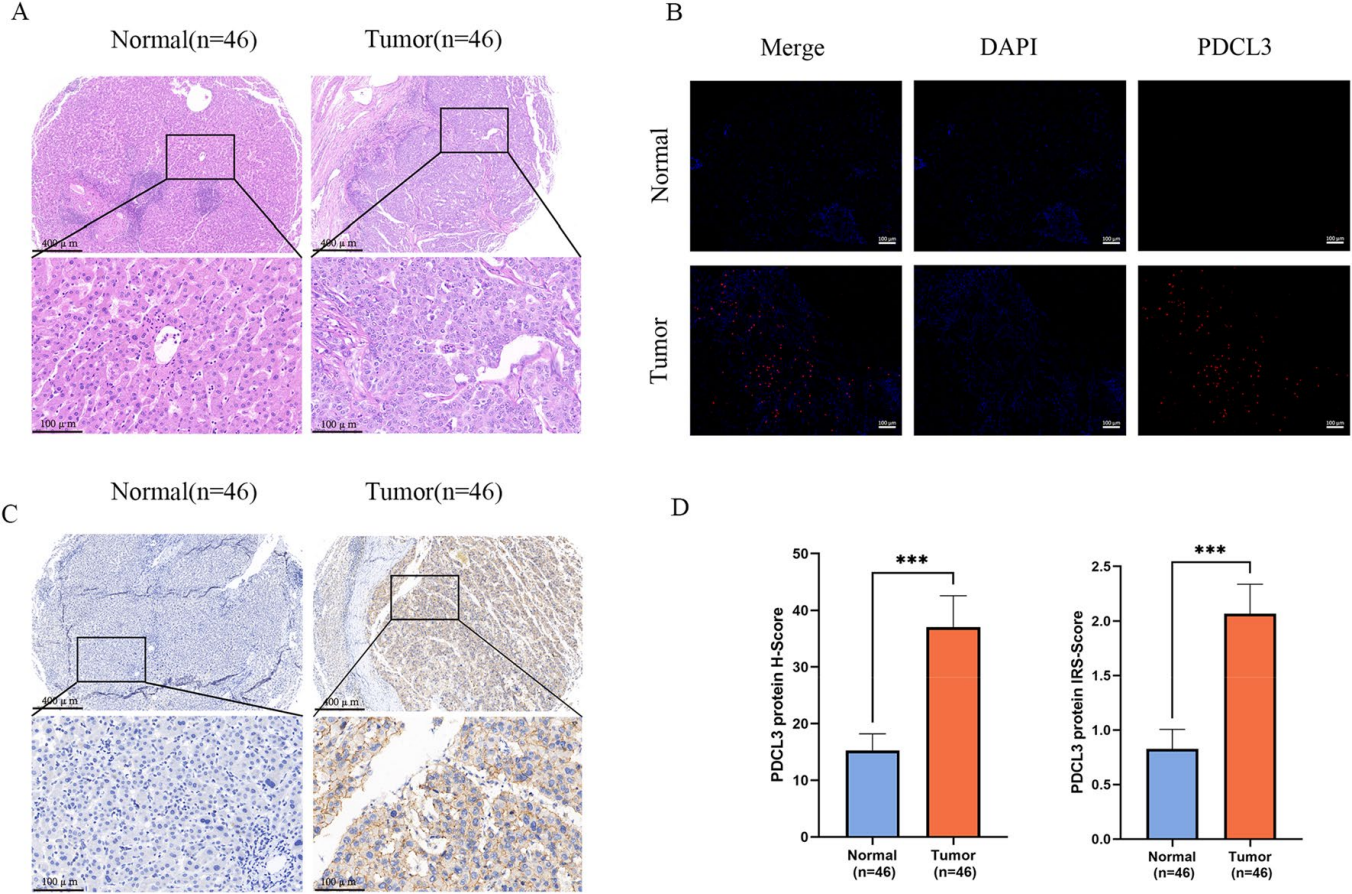
Differential expression of PDCL3 in adjacent non-cancerous tissues and liver cancer tissues. **A** Hematoxylin and eosin (H&E) staining images of normal tissues and liver cancer tissues. **B** Immunofluorescence images depicting the distribution of PDCL3 in normal tissues and liver cancer tissues. **C** Immunohistochemistry staining images showing PDCL3 in normal tissues and liver cancer tissues. **D** H-scores and IRS scores of PDCL3 protein IHC in normal tissues and liver cancer tissues. *p value < 0.05; **p value < 0.01; ***p value < 0.001 (Student’s *t* test)

PDCL3 protein expression in liver cancer and adjacent tissues was examined through immunohistochemistry and immunofluorescence experiments. Hematoxylin and eosin (HE) staining results showed significant morphological differences between liver cancer tissues and adjacent tissues (Fig. 6A). Immunofluorescence experiments confirmed differential distribution of PDCL3 protein in liver cancer tissues and adjacent tissues, with higher PDCL3 protein distribution in liver cancer tissues (Fig. 6B). Immunohistochemical experiments also indicated the same trend, with both IRS scores and H-Score scores showing significantly higher PDCL3 expression levels in liver cancer tissues compared to adjacent tissues (Fig. 6C, D). These results suggest that PDCL3 may play a promoting role in the development of liver cancer.

### *PDCL3 promotes proliferation, migration, invasion, and colony formation of LIHC cells *in vitro

**Fig. 7:**
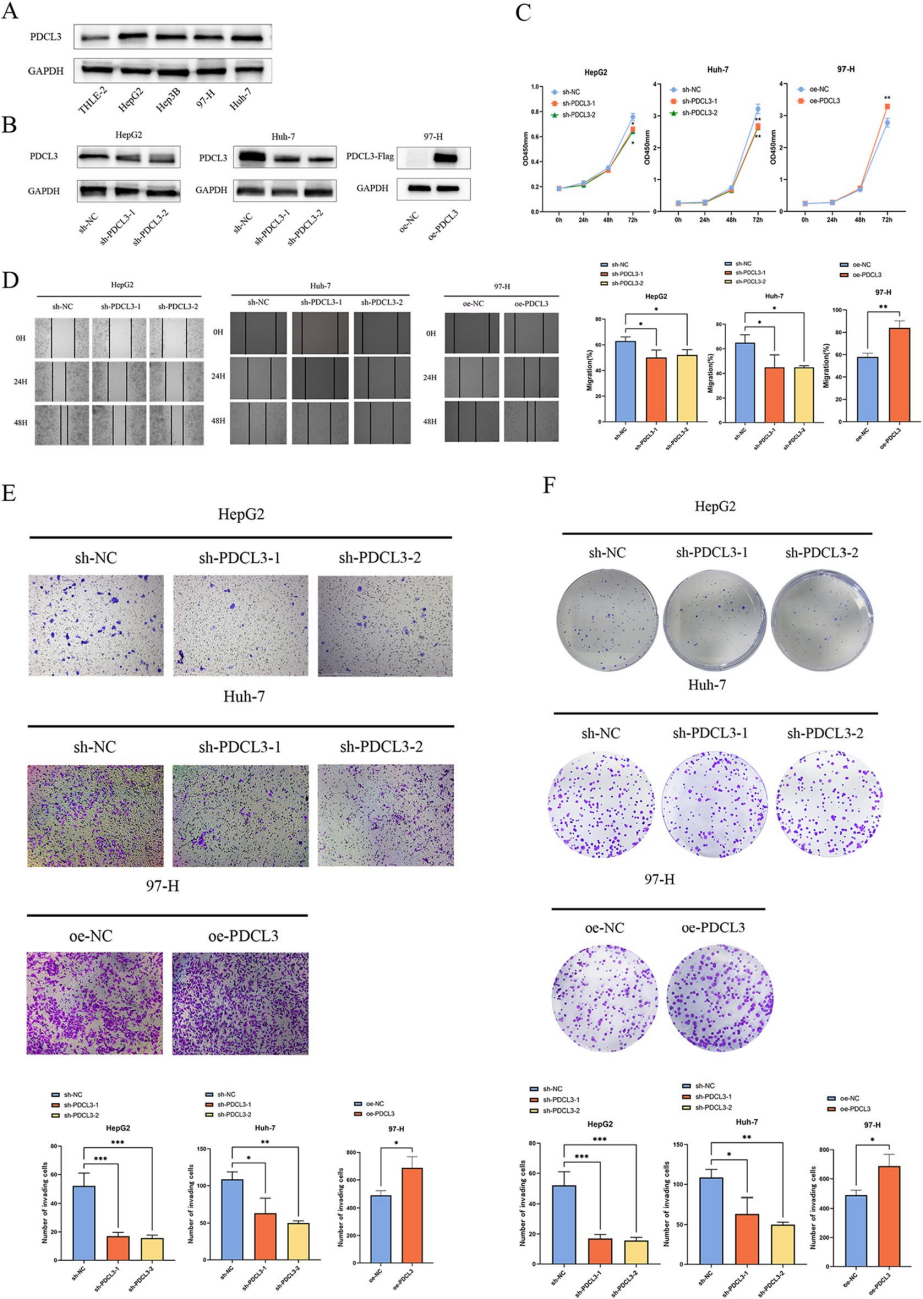
PDCL3 promotes proliferation, migration, invasion, and colony formation capacity of LIHC cells in vitro. **A** Expression levels of PDCL3 in different cell lines. **B** Interference efficiency of PDCL3 detected using the protein imprinting method. **C** Cell proliferation rate determined by the CCK-8 method. **D**, **E** Results and analysis of wound healing and transwell experiments. **F** Results and analysis of colony formation experiment.*p value < 0.05; **p value < 0.01; ***p value < 0.001 (Student’s *t* test)

Based on the results of the protein imprinting experiment (Fig. 7A), we selected HepG2 and Huh-7 cells with relatively higher PDCL3 expression for knockdown treatment, and overexpressed PDCL3 in 97-H cells with relatively lower PDCL3 expression. Subsequently, we utilized protein imprinting experiments to verify the infection efficiency of the virus. (Fig. 7B). The CCK-8 assay was used to evaluate the role of PDCL3 in the growth of LIHC cells. Compared to the control group cells, the proliferation ability of HepG2 and Huh-7 cells with PDCL3 knockdown was significantly inhibited, while the proliferation ability of 97-H cells with PDCL3 overexpression was significantly enhanced (Fig. 7C). These results indicate that PDCL3 is a key regulator of LIHC cell proliferation. Transwell invasion and scratch migration analyses were used to assess the migration ability of LIHC cells with different levels of PDCL3 expression. The results indicate that 97-H cells overexpressing PDCL3 exhibit higher migration ability, while the migration ability of HepG2 and Huh-7 cells with PDCL3 knockdown is significantly reduced (Fig. 7D, E). Subsequently, a colony formation assay was performed to assess the effect of PDCL3 on the colony formation ability of LIHC cells. The results demonstrate that the colony formation capacity is increased in 97-H cells overexpressing PDCL3, while the colony formation ability of HepG2 and Huh-7 cells with PDCL3 knockdown is lower than that of the control group (Fig. 7F). These experiments indicate that PDCL3 can promote the proliferation, migration, invasion, and colony formation ability of LIHC cells in vitro.

## Discussion

Opsin-like proteins constitute a conserved protein family featuring a thioredoxin-like domain, encompassing Phosducin-like protein 1 (PhLP1), Phosducin-like protein 2A (PDCL3), Phosducin-like protein 2B (PDCL3), and Phosducin-like protein 3 (PhLP3) [11–13]. Initially discovered in the retina and pineal gland, PDCL3 forms a complex with the βγ subunit of retinal G-proteins, playing a pivotal role in the visual system and is thus termed a photoreceptor [14]. Subsequent research unveiled its presence in the cytoplasm and endoplasmic reticulum, where it acts as a chaperone protein interacting with VEGFR-2, safeguarding it from misfolding, aggregation, and reducing ubiquitination-induced degradation [15, 16]. Consequently, PDCL3-mediated reduction in VEGFR-2 degradation may significantly contribute to rapid tumor growth. In the eukaryotic cytoplasm, chaperone protein CCT facilitates the folding and assembly of various substrate proteins involved in cellular cytoskeletal components, including actin, Cdc20p, Cdc50p, and other yet-to-be-confirmed substrates. Studies indicate PDCL3's ability to form a ternary complex with CCT and its substrates, thereby regulating substrate protein folding, influencing cytoskeletal formation, and the cell cycle [13, 17]. PDCL3 also exhibits chaperone protein function through its interaction with heat shock protein Hsp-90, mitigating the unfolding and aggregation of target proteins under stress conditions, akin to Hsp-90 and Hsp-70, thus reducing stress-induced damage [18, 19].

In this study, we initially analyzed PDCL3 expression across various tumors. The TIMER database reveals significantly increased PDCL3 expression in BRCA, CHOL, COAD, ESCA, HNSC, LIHC, LUAD, LUSC, PRAD, STAD, and UCEC, while it is markedly reduced in KICH and PCPG. Analysis of the CPTAC database suggests slight variations but overall indicates differential PDCL3 expression in multiple tumors. Furthermore, we explored the association between PDCL3 and clinical prognosis. Pan-cancer analysis from GEPIA and TCGA databases demonstrates that elevated PDCL3 expression correlates with poorer outcomes in ACC, KICH, LGG, LUAD, LIHC, MESO, and UVM. In summary, our findings indicate that high PDCL3 expression predicts poorer overall survival (OS) and may serve as a potential prognostic marker for cancer.

LIHC currently ranks among the most common malignant tumors. Recent statistics place LIHC sixth in terms of incidence and third in terms of mortality, primarily due to its high metastatic rate and rapid malignant progression [20]. The pathogenesis and risk factors of LIHC are multifaceted, and the molecular mechanisms underlying its development remain elusive. Hepatitis, aflatoxin, metabolic syndrome, and genetic factors are suggested as LIHC risk factors [21–26]. Ultrasound (US) and serum alpha-fetoprotein (AFP) are commonly used clinical methods for LIHC diagnosis, albeit lacking specificity and sensitivity [27]. Therefore, novel biomarkers for early LIHC diagnosis are a recent research focus.

In our pan-cancer data analysis, we observed a close association between PDCL3 and LIHC. Subsequently, we conducted further research to investigate PDCL3's functional role in LIHC. Initially, we examined PDCL3 expression in different subgroups, observing higher expression in clinical, pathological, and tissue stages associated with poorer outcomes. Immunohistochemistry (IHC) images from the HPA database depicted higher PDCL3 expression in tumor tissues. Notably, the ROC curve area (AUC) for diagnosing LIHC using PDCL3 reached 0.944. PDCL3 expression levels effectively predicted 1-year and 3-year overall survival rates for patients. Therefore, PDCL3 emerges as a potential biomarker for LIHC diagnosis.

Immunotherapy stands out as an effective treatment for advanced LIHC [28, 29]. The LIHC microenvironment, primarily composed of tumor cells and immune cells [30–32], presents complexity and dynamics, leading to reduced immunotherapy effectiveness due to drug resistance or immune escape [33], necessitating further research on new immune-related therapeutic targets [34].

Through PDCL3 study in LIHC, we observed significant enrichment of biological activities related to immune infiltration, such as humoral immune response, immunoglobulin-mediated immune response, B-cell-mediated immunity in the biological process (BP), immunoglobulin complexes, circulating immunoglobulin complexes in the cellular component (CC), and immunoglobulin receptor binding in the molecular function (MF). These findings suggest PDCL3's association with the immune system. Using the TIMER database, we explored the relationship between PDCL3 and immunity, revealing a negative correlation between PDCL3 expression and macrophages (Rho = − 0.481, *p* = 2.13e − 21), but no significant correlation with other immune cells. Further survival analysis indicated that patients with high PDCL3 expression in tumors and low macrophage infiltration had a poorer prognosis, partly attributed to reduced macrophage infiltration. Previous research on immune infiltration in glioblastomas suggested that M2-type macrophage infiltration influences glioblastoma patient prognosis through angiogenesis regulation, extracellular matrix remodeling, and glioblastoma cell invasiveness [35]. Therefore, we hypothesize that high PDCL3 expression in LIHC affects the biological behavior of tumors by reducing macrophage infiltration. Additionally, we found a positive correlation between PDCL3 and immune checkpoint molecules, including CD274, CTLA4, HAVCR2, PDCD1, and TIGIT. Therefore, PDCL3 may serve as a relevant target in immunotherapy. Previous studies indicated that LIHC patients with high macrophage infiltration have a better prognosis [36], consistent with our findings.

Based on the aforementioned bioinformatics analysis, we collected pathological samples from clinical LIHC patients to construct tissue microarrays for PDCL3 expression validation in liver cancer. Immunofluorescence and immunohistochemistry experiments indicated significantly higher PDCL3 protein expression levels in liver cancer compared to adjacent normal tissues, consistent with PDCL3 expression analysis results in public databases. This suggests that PDCL3 may play a promoting role in liver cancer occurrence and development. To further confirm PDCL3’s role in liver cancer development, we modulated PDCL3 gene expression in liver cancer cells via retroviral transfection and investigated PDCL3’s impact on liver cancer cell biological behavior. CCK-8 assays, scratch wound healing experiments, transwell assays, and colony formation assays revealed that PDCL3 significantly promotes tumor cell proliferation, migration, invasion, and colony-forming ability, aligning with our bioinformatics predictions and confirming PDCL3 as an oncogenic gene in LIHC.

## Conclusion

In summary, employing bioinformatics techniques, our research underscores PDCL3’s high expression in various tumors, associating it with poor prognosis. High PDCL3 expression in LIHC correlates with prognosis and serves as a potential diagnostic biomarker. PDCL3’s association with immune infiltration may influence LIHC cellular behavior by reducing. Finally, although this study reveals PDCL3 as a novel prognostic marker for liver cancer from multiple perspectives, further exploration is needed to understand its mechanisms and regulatory details.

## Data Availability

The data underlying this article will be shared on reasonable request to the corresponding author.

## Abbreviations

PDCL3: Phosducin-like 3
PhLPs: Phosducin-like proteins
HCC: Hepatocellular carcinoma
VEGF: Vascular endothelial growth factor
HE: Hematoxylin–eosin
IHC: Immunohistochemistry
IF: Immunofluorescence
CCK-8: Cell counting kit-8
TCGA: The Cancer Genome Atlas
TIMER: Tumor Immunity Evaluation Resource
GEPIA: Gene Expression Profiling Interactive Analysis
OS: Overall survival
PFS: Progression-free survival
RFS: Relapse-free survival
DSS: Disease-specific survival
ROC: Receiver operating characteristic
IHC: Immunohistochemistry
BRCA: Breast invasive carcinoma
LGG: Brain lower grade glioma
LIHC: Liver hepatocellular carcinoma
LUAD: Lung adenocarcinoma
PRAD: Prostate adenocarcinoma
STAD: Stomach adenocarcinoma
UCEC: Uterine corpus endometrial carcinoma
TMB: Tumor mutational burden
GO: Gene Ontology
KEGG: Kyoto Encyclopedia of Genes and Genomes
GSEA: Gene Set Enrichment Analysis
TPM: Transcripts per million
KM: Kaplan–Meier
CHOL: Cholangiocarcinoma
COAD: Colon adenocarcinoma
ESCA: Esophageal cancer
HNSC: Head and neck squamous cell carcinoma
LUSC: Lung squamous cell carcinoma
KICH: Kidney chromophobe
PCPG: Pheochromocytoma and paraganglioma
DLBC: Diffuse large B-cell lymphoma
GBM: Glioblastoma
PAAD: Pancreatic adenocarcinoma
TGCT: Testicular germ cell tumor
THYM: Thymoma
ccRCC: Clear cell renal cell carcinoma
ACC: Adrenocortical carcinoma
MESO: Mesothelioma
UVM: Uveal melanoma
HR: Hazard ratio
CI: Confidence interval
KICH: Kidney chromophobe
KIRP: Kidney renal papillary cell carcinoma
THCA: Thyroid carcinoma
KIRC: Kidney clear cell carcinoma
SKCM: Skin cutaneous melanoma
BLCA: Bladder cancer
